# Instability of the proximal radioulnar joint in Monteggia fractures—an experimental study

**DOI:** 10.1186/s13018-019-1367-7

**Published:** 2019-11-28

**Authors:** Achim Biewener, Fabian Bischoff, Tobias Rischke, Eric Tille, Ute Nimtschke, Philip Kasten, Klaus-Dieter Schaser, Jörg Nowotny

**Affiliations:** 1 Orthopaedic-Traumatology Centre (OUC), Carl Gustav Carus University, Technical University Dresden, Fetscherstraße 74, 01307 Dresden, Germany; 2 Institute of Anatomy, Carl Gustav Carus University, Technical University Dresden, Dresden, Germany; 3 Orthopaedic-Surgery Centre (OCC), Tübingen, Germany; 4 Centre for Translational Bone, Joint and Soft Tissue Research, Technical University Dresden, Dresden, Germany

**Keywords:** Elbow, Monteggia fracture, Proximal radioulnar joint, Radial head dislocation, Biomechanical study, Annular ligament, Interosseous membrane, Ligament reconstruction

## Abstract

**Background:**

A Monteggia fracture is defined as a fracture of the proximal ulna combined with a luxation of the radial head. The aim of the present work is to evaluate the extent of instability of the radius head in the proximal radioulnar joint (PRUJ) as a function of the severity of elbow fracture and ligamentous injury in an experimental biomechanical approach.

**Methods:**

Eight fresh-frozen cadaver arms were used. All soft tissues were removed except for the ligamentous structures of the PRUJ and forearm. A tensile force of 40 N was exerted laterally, anteriorly or posteriorly onto the proximal radius. The dislocation in the PRUJ was photometrically recorded and measured by two independent examiners. After manual dissection of the ligamentous structures up to the interosseous membrane, the instability was documented and subsequently measured. The following dissection levels were differentiated: intact ligamentous structures, dissection of annular ligament, oblique cord and proximal third of interosseous membrane.

**Results:**

An anterior instability remains relatively constant until the proximal third of the interosseous membrane is dissected. The radial head already dislocates relevantly in the posterior direction after dissection of the annular ligament with an additional considerable stability anteriorly and laterally. Subsequently, the posterior instability increases less pronouncedly in regard of distal resected structures. The lateral instability increases constantly during the progressing resection of the ligamentous structures.

**Conclusion:**

On the one hand, a complete healing of the ligament injury after functional treatment is hardly conceivable with ligamentary damage up to the level of the proximal interosseous membrane. A remaining instability of the proximal radius could therefore be a possible cause for the unsatisfactory clinical results after certain Monteggia fractures. On the other hand, the present study may give a possible explanation (i.e. early dorsal radius head dislocation after dissection of annular ligament) why the Bado II injury is the most frequent type of Monteggia fractures.

## Introduction

A Monteggia fracture is defined as a fracture of the proximal ulna combined with a dislocation of the radial head [1]. The current operative treatment of these injuries leads to favourable clinical results in the majority of cases. However, there are some injuries that do not have a favourable outcome. The knowledge of the fracture morphology and its involved structures are therefore important preconditions for a successful therapy [2].

Monteggia fractures are usually associated with a dislocation in the proximal radioulnar joint (PRUJ) [2, 3]. The most commonly used classification according to Bado describes in four subtypes the direction of the radius head dislocation and thus the angulation of the ulna fracture [4]. The posterior Monteggia injury (Bado type II) is additionally classified according to Jupiter into four subtypes and describes the accompanying ulna fracture or radius head injury [5]. The associated extent of the capsule-ligament injury can only be assumed. While during childhood the injury often heals with very good results, a complicative healing process is often observed for injuries in adults [6].

**Fig. 1:**
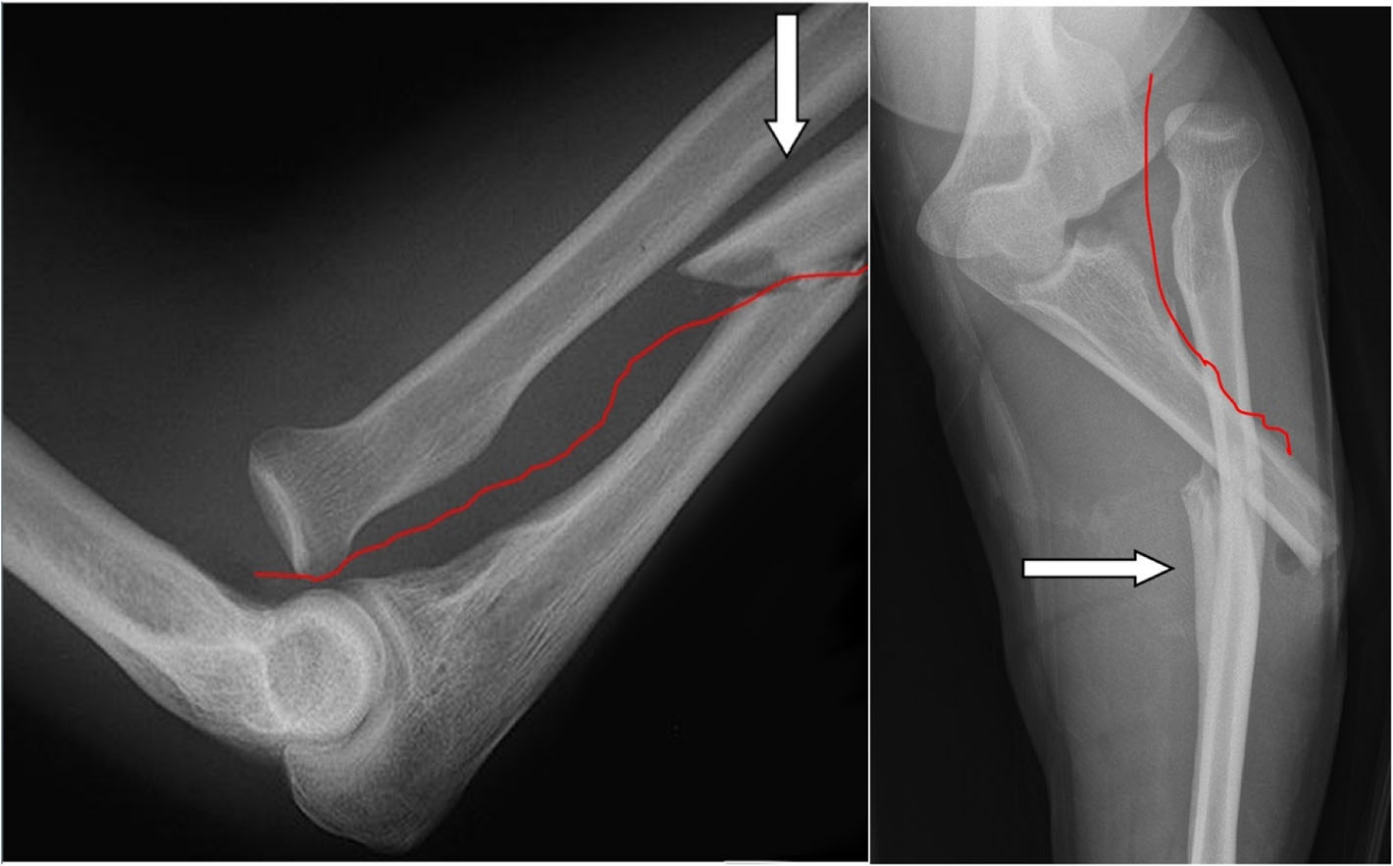
X-ray of a Monteggia fracture (Bado I) with potential injury of the proximal ligamentary structures between the ulna and the radius including the PRUJ until the end of the distal ulnar fracture (white arrow) in left: lateral and right: anteroposterior view

**Fig. 2:**
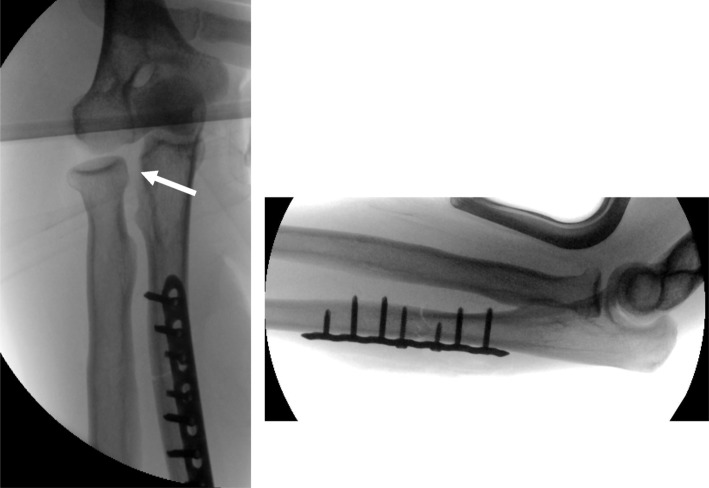
Intraoperative X-ray after plate osteosynthesis of a Monteggia fracture with remaining instability in the PRUJ (white arrow)

There is an agreement in the literature that the precise anatomical reconstruction of the ulna fracture is the key to a successful surgical therapy [7]. In general, the distal part of the ulna fracture that remains intact in the interosseous membrane leads to a reduction of the radius head in the elbow joint or in the PRUJ. In order to dislocate the radius head with intact capsule ligament structures of the humeroulnar joint part, the ligament connections between radius and ulna (consisting of annular ligament, chorda obliqua and proximal part of the interosseous membrane) must rupture at the level of the ulna fracture (Fig. 1). The distal part of the interosseous membrane of the fracture usually remains intact. Even after anatomical reduction and fixation of the ulna fracture, a persistent instability auf the PRUJ can remain (Fig. 2).

It is not known whether the torn ligament connections between the radius and ulna actually heal to a stable condition without surgical revision and under the obligatory early functional treatment. A standard surgical refixation of the torn annular ligament is not recommended [2, 8]. However, it can be assumed that with ulnar osteosynthesis alone, an instability of the radius head will remain in the PRUJ and in relation to the humeral capitulum. It can be assumed that their extent increases significantly from proximal to distal depending on the level of the ulna fracture.

The aim of the present work is to evaluate the extent of instability of the radius head in the PRUJ as a function of the severity of elbow fracture and ligament injury in an experimental and biomechanical approach.

## Methods

### Specimens

Eight fresh-frozen cadaver arms were provided by the Institute of Anatomy of the university clinic, Technical University Dresden, Germany. The specimens were frozen at − 22 °C (Liebherr Typ 40073 1, Germany). Within the preparation, all soft tissues were removed except for the ligament structures of the PRUJ and forearm. The distal ulna was solid clamped in a vise. To reduce stability variation, the preparation was carried out by a single senior orthopaedic surgeon in a standardized fashion. Todisco and Trisi had already proven that Hounsfield units (HU) measured in CT correlate highly with bone mineral density [9]. Therefore, the bone density of the specimens was measured by using a quantitative computed tomography (Somatom CT, Siemens, München, Germany, technical specifications: CTDI 4.53 vol mGy, kV 80, mAs 180, 0.75 mm layer thickness). The bone density of all used proximal ulnas were in average 596 ± 127 (min 495, max 891) HU.

### Test setup and intervention

A 4.5-mm Schanz screw was inserted in a vertical direction and perpendicular in the horizontal plane. Clockwise markings at 3, 6 and 9 o’clock were applied to the radius head. Furthermore, the lowest point in the PRUJ was marked as a reference (Fig. 2). By means of a mechanical force measuring device (PGH, Kraftmessgeraete, Halle [Saale], Germany), a tensile force of 40 N was exerted laterally, anteriorly or posteriorly on the proximal radius. The dislocation in the PRUJ was photometrically recorded and measured by two independent investigators using image processing software (Paint.net, dotPDN LLC).

**Fig. 3:**
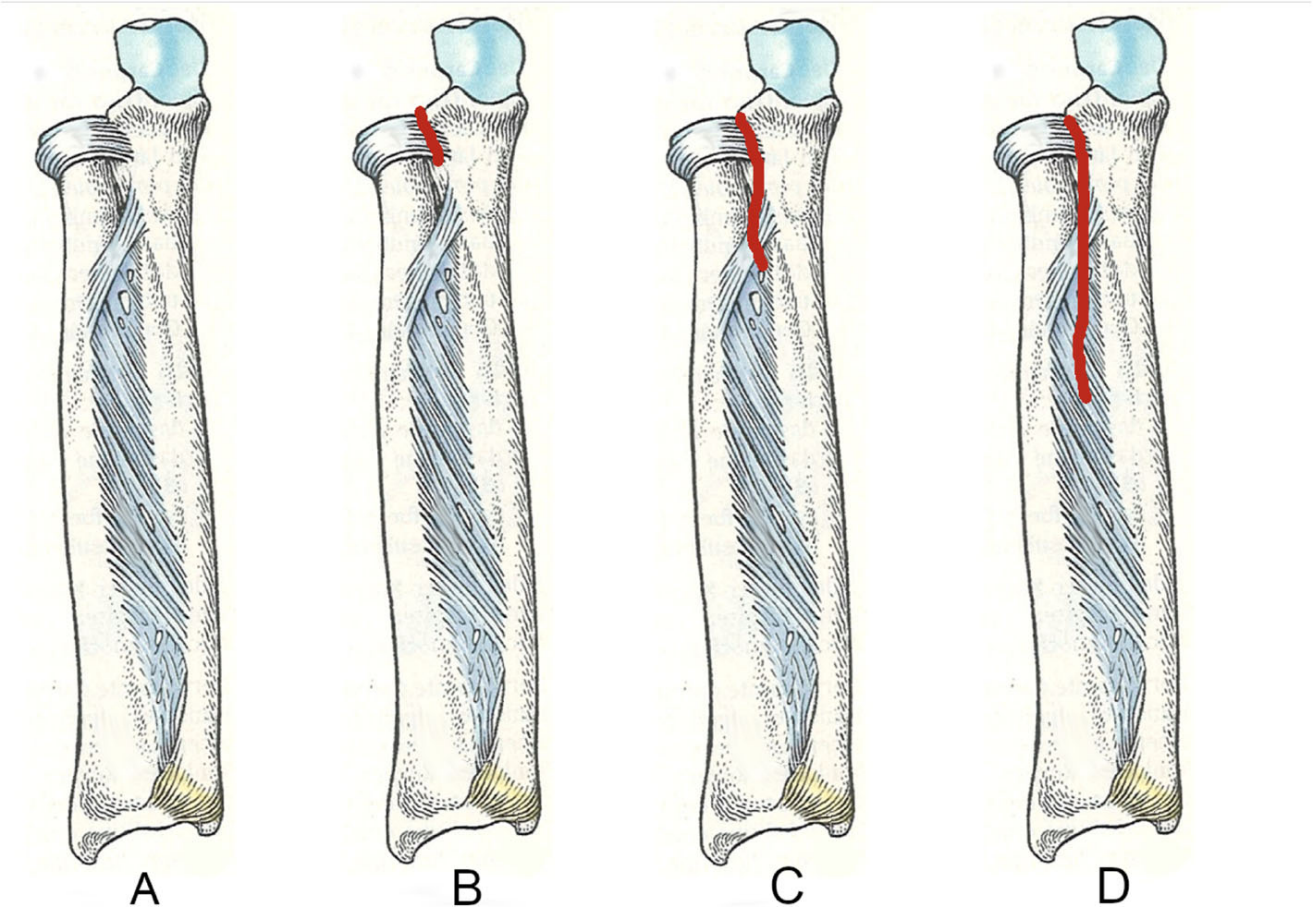
Drawing of the forearm with the level of the dissection (red line). **a** Intact ligament structures. **b** Annular ligament. **c** Annular ligament and oblique cord. **d** Annular ligament, oblique cord and proximal third of interosseous membrane

After manual dissection of the ligament structures up to the interosseous membrane, the instability was documented and subsequently measured. The following dissection levels were differentiated (Fig. 3):

A. Intact ligament structures
B. Dissection of annular ligament
C. Additional dissection of the annular ligament and oblique cord
D. Additional dissection of annular ligament, oblique cord and proximal third of interosseous membrane

Statistical analysis was performed with SPSS Statistics software (version 25; IBM, Armonk, NY, USA) for descriptive statistics. The significance level was chosen at *p* < 0.05. All data are presented as mean with standard deviation, minimum and maximum. Univariate analysis of variance was carried out to compare the different instabilities.

## Results

The average age of the used donors was 81.6 ± 9.4 (62–92) years. Five donors were female and three male. All biomechanical tests were successfully completed without the Schanz screws or the holding device loosening itself.

### Setting A (intact ligament structures)

With intact ligament structures there is almost no instability in the PRUJ. It measures 1.5 mm (SD 1.08, min 0, max 2.7) in the anterior direction, 0.7 mm (SD 1.28, min 0, max 3.0) in the lateral direction and 1.6 mm (SD 1.57, min 0, max 3.9) in the posterior direction. There was no significant difference among these groups.

### Setting B (dissection of annular ligament)

**Fig. 4:**
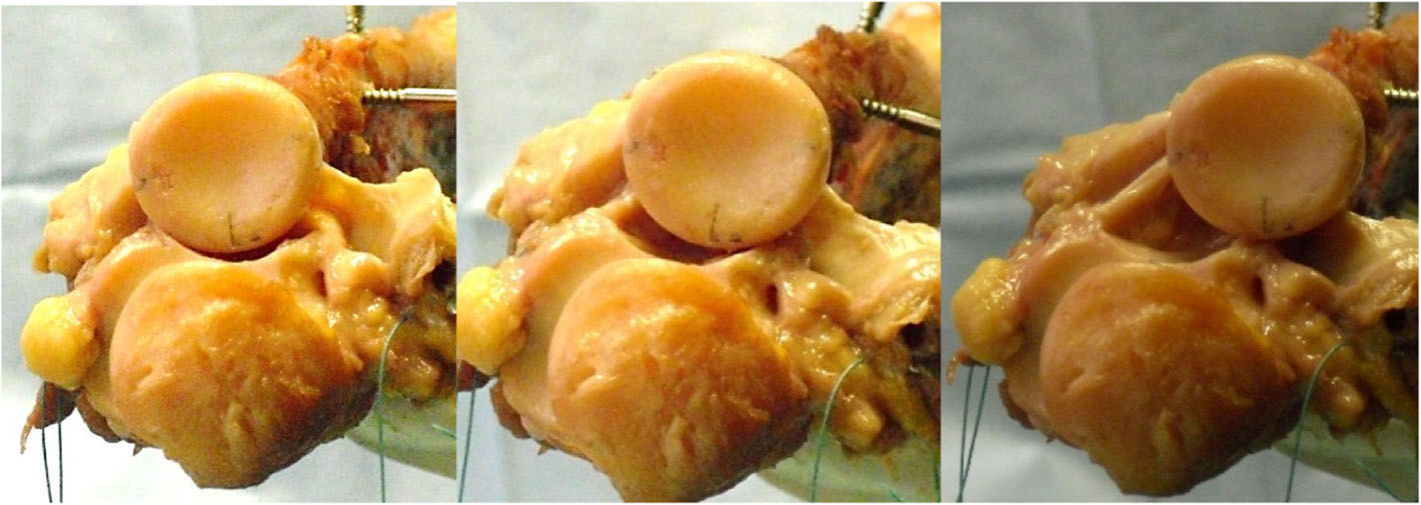
Instability of the PRUJ dissection of the annular ligament (left: anterior, centre: lateral, right: posterior)

After dissection of the annular ligament, instability occurs mostly posteriorly and slightly laterally. An anterior instability is almost not measured. An instability of 1.8 mm (SD 1.7, min 0, max 4.2) in the anterior direction, 4.1 mm (SD 2.7, min 2.4, max 10.1) in the lateral direction and 13.9 mm (SD 4.05, min 10.8, max 22.9) in the posterior direction was recorded (Fig. 4).

### Setting C (dissection of the annular ligament and oblique cord)

After dissection of the annular ligament and oblique cord, another posterior instability is generated. A lateral instability of 5.7 mm (SD 2.3, min 2.5, max 9.72), an anterior instability of 2.9 mm (SD 1.7, min 0, max 1.7) and a posterior instability of 17.5 mm (SD 6.3, min 10.5, max 26.5) were measured.

### Setting D (dissection up to proximal third of interosseous membrane)

After the dissection of the proximal third of the interosseous membrane, a massive multidirectional instability was observed in the lateral direction with dislocation of the radius head in the PRUJ in the posterior and anterior direction. In detail, there was a lateral instability of 10.3 mm (SD 2.6, min 6.7, max 14.2), an anterior instability of 15.8 mm (SD 5.3, min 9.2, max 23.1) and a posterior instability of 23.9 mm (SD 12, min 10.1, max 45.2).

### Instability in regard of direction

**Fig. 5:**
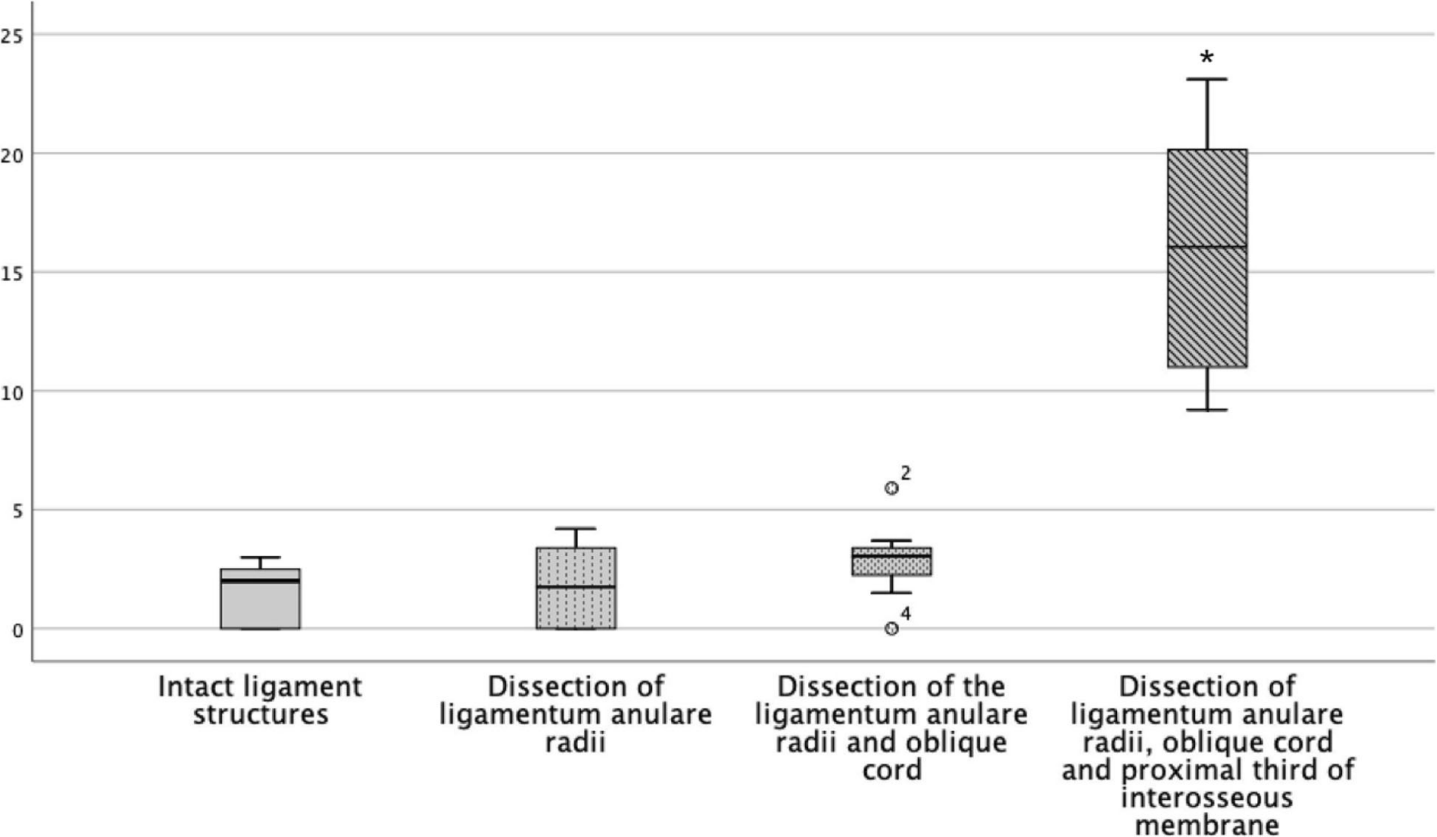
Boxplot of instability in millimetres of the radius head in the anterior direction

**Fig. 6:**
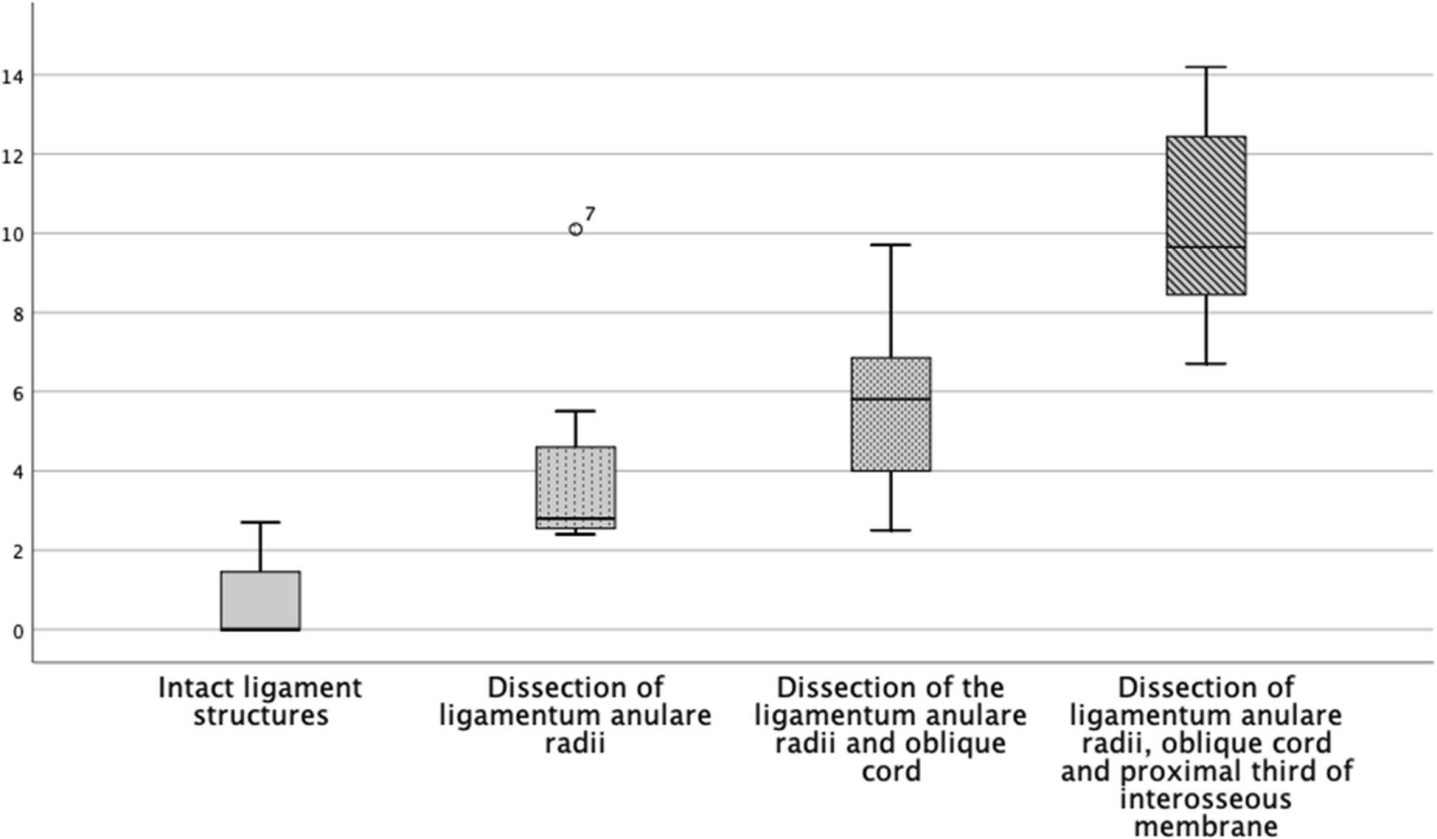
Boxplot of instability in millimetres of the radius head in the lateral direction

**Fig. 7:**
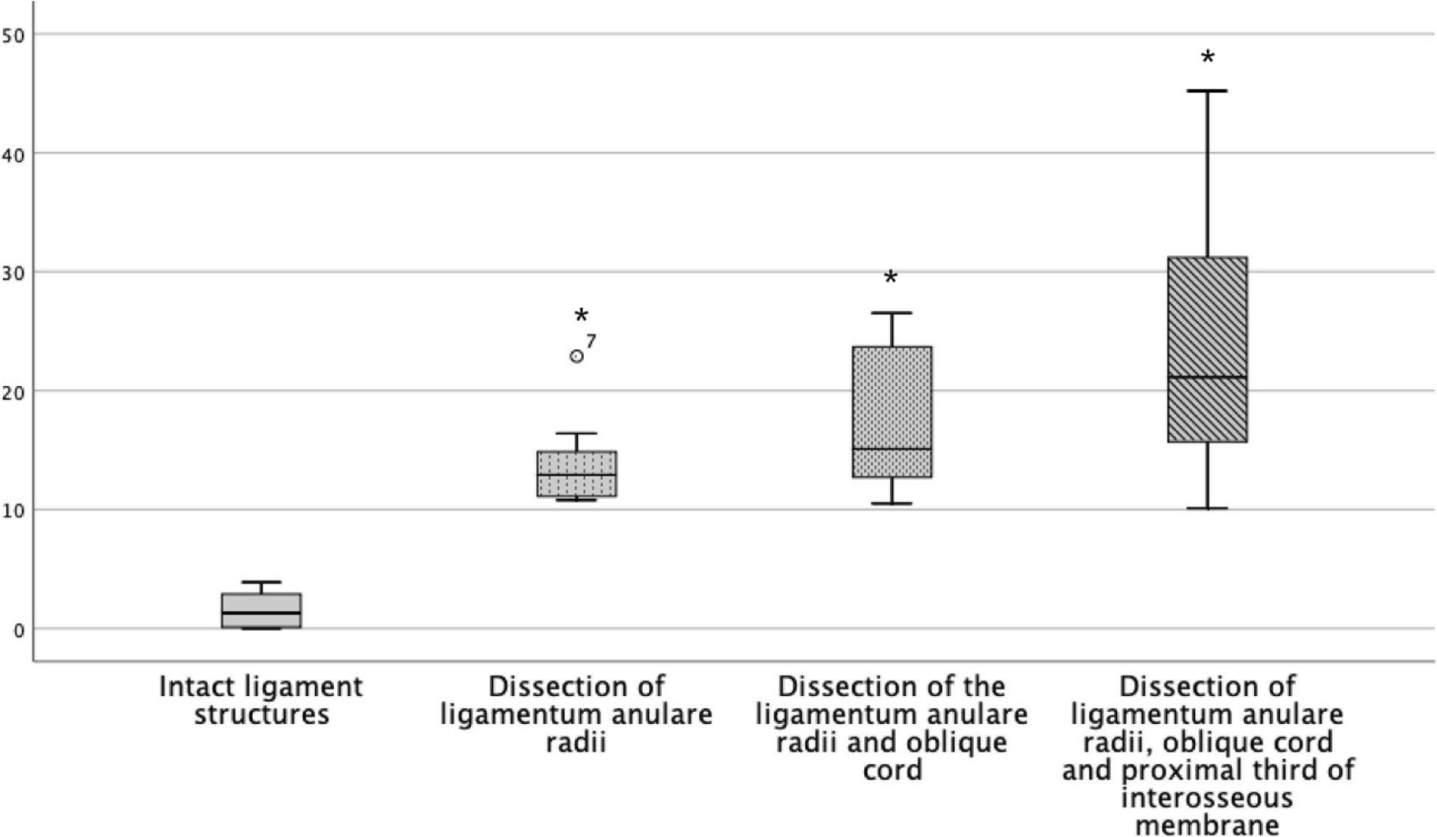
Boxplot of instability in millimetres of the radius head in the posterior direction

Considering the instability in regard of the direction, it is noticeable that the anterior instability remains relatively constant until the proximal third of the interosseous membrane is dissected (Fig. 5). This is also seen statistically with a significant increase of the instability when dissecting the interosseous membrane [*p* = .001]. The early subluxation of the radius head in the posterior direction after dissection of the annular ligament with considerable stability to anterior and lateral is remarkable. In the course of our examinations, the posterior instability increased in inverse proportion with initial large increase of instability and decreases in regard of the distally resected structures (Fig. 7). However, the successive instability is always significant (Table 1). The lateral instability increases relatively constantly during the resection of the ligament structures distally. It is striking that a slight translation to the posterior direction always occurs with lateral traction. However, only the lateral offset was measured (Fig. 6).

**Table 1:** Overview of the measurement data

Direction of dislocation	Involved structure	Instability in millimetres	Min	Max	SD	*p*
Anterior	Intact	0.5	0	2.7	1.08	
	Annular ligament	1.8	0	4.2	1.7	.699
	Oblique cord	2.9	0	1.7	1.7	.219
	Interosseous membrane	15.8	9.2	23.1	5.3	.000
Lateral	Intact	0.7	0	3.0	1.28	
	Annular ligament	4,1	2.4	10.1	2.7	.002
	Oblique cord	5.7	2.5	9.72	2.3	.031
	Interosseous membrane	10.3	6.7	14.2	2.6	.000
Posterior	Intact	1.6	0	3.9	1.57	
	Annular ligament	13.9	10.8	22.9	4.05	.000
	Oblique cord	17.5	10.5	26.5	6.3	.047
	Interosseous membrane	23.9	10.1	45.2	12	.045

## Discussion

Precise ligamentous guidance of the radius rotating around the ulna is essential for free range of motion and painless strength of the forearm. The translation of the radius head during forearm rotation is therefore limited to only 1–2 mm for intact ligaments between the ulna and the radius bone [10, 11]. In case of Monteggia fractures, besides the anatomical reconstruction of the ulna fracture, the objective of the treatment must be the sufficient healing of the ligamentous structures in the PRUJ and the interosseous membrane.

In the literature, only three studies investigate experimentally the resulting instability in the PRUJ after cutting band structures [12–14]. All of these studies have evaluated the effect of ligamentary structure resection in regard of the stability in the PRUJ. In the study according to Galik et al., the translation of the radius head increased from 1.6 ± 0.7 to 2.3 ± 0.9 mm in the mediolateral (ml) plane and from 2.1 ± 0.6 to 2.6 ± 0.9 mm in the anteroposterior (ap) plane after severing the annular ligament during pro-/supination [12]. A direct comparison to the present study is difficult because only the sum of the distance in one plane (ap and ml) was measured without the exact data for the anterior, lateral or posterior plane being given. In this study, however, the complete elbow joint in the 90° position with intact lateral collateral ligament was tested, which also makes the comparability difficult, because the 90° position of the elbow is a very stable position anyway when the primary stabilizing ligaments were not resected.

A comparable experimental setup has been chosen in the study of Anderson et al. The forearm including the elbow joint was examined and the ulnar collateral ligament, the lateral ulnar collateral ligament (LUCL) and the joint capsule of the elbow were left intact during preparation [13]. After dissection of the annular ligament, chorda obliqua and proximal interosseous membrane, the dislocation of the radius head in the PRUJ was measured in the lateral direction. Even after the dissection of all structures except for the distal interosseous membrane, the maximum diameter was only 3 (SD 2) mm. Due to the intact primary ligamentary structures, the study is difficult to compare with the present study. However, there is no relevant instability in any direction in the PRUJ, which indicates in comparison to our study, that the not resected structures (ulnar collateral ligament, the LUCL and the joint capsule) contribute to considerable stability. In the present study, the instability of the PRUJ was therefore measured just by the use of forearm specimens without an annexed elbow joint and after resection of the medial und lateral ligament structures.

The resulting instability of the radius head was more obvious in the experimental approach of Galik et al. [12]. The elbow joint with capsule and ligament structures remained intact and the specimen was clamped in 90° elbow flexion. The dislocation of the radius head in lateral, anterior and posterior plane after application of 20 N tensile force was measured and reported in percent of the diameter to the radius head. After dissection of the annular ligament, a significant lateral (46%) and posterior (37%) instability was measured, while stability in the anterior direction (8%) was retained. The same results were seen in the present study with no significant instability in the anterior direction and already subluxation of the radius head in the lateral and posterior direction. However, in the study of Hayami et al., it was larger in the lateral direction, while in the present study, the largest instability was evaluated in the posterior direction after dissection of the annular ligament [14].

Not until the separation of the proximal half of the interosseous membrane, a subluxation was observed in the anterior direction (39%) and even further in the lateral (154%) and posterior (200%) direction. In comparison to the present study, these results correspond precisely to the currently evaluated data. Also in the present study, a dislocation in the PRUJ in the lateral and posterior plane was evaluated significantly after resection up to the membrane interossea, whereas in the anterior direction, only a comparatively low dislocation was found. However, the results of these experimental studies can only be transferred to a very limited degree onto the instability of the PRUJ after Monteggia fractures. In particular, in the 90° elbow flexion with intact collateral ligaments, the guidance of the concave radius head on the convexity of the humeral capitulum may result in a considerable secondary stability in the frontal and sagittal planes. The dislocation of the radius head often leads to significant ruptures of the elbow joint capsule and the radial collateral ligament complex, so that articular guidance of the radius head is not possible even after stable ulna osteosynthesis (Fig. 2).

The study has some limitations. On the one hand, in the present study, a different experimental setup was chosen (no 90° position of the elbow) and the primary and secondary stabilizing structures such as the collateral ligaments and joint capsule with the distal humerus were resected. However, we believe that a stability bias is created by the very stable 90° position of the elbow, especially since the relevant instabilities of the elbow are created starting at approximately 30° extension. On the other hand, compared to Hayami et al., we measured with double the force (20 vs. 40 N), so in the present study, the measured instability is higher compared to other studies [14]. Nevertheless, we believe that 40 N is more appropriate in relation to the forearm natural weight. A further limitation is the analogue, manual measurement of the instability by an image processing software, which can result in a latent inaccuracy. However, we have tried to reduce this by using two independent investigators. A measurement with an optical system would be preferable for future studies.

## Conclusion

Based on our experimental observation and the study of Hayami et al., a complete healing of the instability of the radial head under functional treatment is hardly conceivable at least for ligamentous injuries up to the chorda obliqua or proximal interosseous membrane. A remaining instability of the proximal radius is a possible cause for the unsatisfactory clinical results after certain Monteggia fractures. Therefore, we recommend an intraoperative stress test of the PRUJ (equivalent to the syndesmosis stress testing) after anatomically stable osteosynthesis of the ulna, and, in case of persisting significant instability, an operative reconstruction of the annular ligament.

In addition, the present study may give a possible explanation (i.e. early dorsal radius head dislocation after dissection of annular ligament) why the Bado II injury is the most frequent type of Monteggia fractures.

## Data Availability

The material and the data are made available.

## Abbreviations

ap: Anteroposterior
CT: Computer tomography
Fig: Figure
HU: Hounsfield unit
LUCL: Lateral ulnar collateral ligament
PRUJ: Proximal radioulnar joint
SD: Standard deviation
